# Matching Rules for Collective Behaviors on Complex Networks: Optimal Configurations for Vibration Frequencies of Networked Harmonic Oscillators

**DOI:** 10.1371/journal.pone.0082161

**Published:** 2013-12-26

**Authors:** Meng Zhan, Shuai Liu, Zhiwei He

**Affiliations:** 1 Wuhan Center for Magnetic Resonance, State Key Laboratory of Magnetic Resonance and Atomic and Molecular Physics, Wuhan Institute of Physics and Mathematics, Chinese Academy of Sciences, Wuhan, China; 2 University of the Chinese Academy of Sciences, Beijing, China; Technical University of Madrid, Italy

## Abstract

The structure-dynamics-function has become one of central problems in modern sciences, and it is a great challenge to unveil the organization rules for different dynamical processes on networks. In this work, we study the vibration spectra of the classical mass spring model with different masses on complex networks, and pay our attention to how the mass spatial configuration influences the second-smallest vibrational frequency (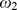) and the largest one (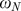). For random networks, we find that 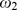 becomes maximal and 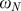 becomes minimal if the node degrees are point-to-point-positively correlated with the masses. In these cases, we call it point-to-point matching. Moreover, 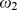 becomes minimal under the condition that the heaviest mass is placed on the lowest-degree vertex, and 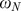 is maximal as long as the lightest mass is placed on the highest-degree vertex, and in both cases all other masses can be arbitrarily settled. Correspondingly, we call it single-point matching. These findings indicate that the matchings between the node dynamics (parameter) and the node position rule the global systems dynamics, and sometimes only one node is enough to control the collective behaviors of the whole system. Therefore, the matching rules might be the common organization rules for collective behaviors on networks.

## Introduction

Various dynamical processes on complex networks [1]–[16] have attracted a great deal of interest in many disciplines, ranging from physical, chemical, and biological sciences, to even social sciences and engineering technology, with the central target to decipher and even utilize the emergent features coming out of the interplay between the structure and function via dynamics. Since a function is a dynamic property, the structure-dynamics-function has been one of common problems in modern sciences [1]–[5]. These dynamical behaviors can be very rich including synchronization in coupled nonlinear systems, consensus or grouping in multi-agent systems, self-organized criticality, epidemic spreading, traffic jam, stochastic resonance, and many others. They pose astonishing challenges for us to unveil the organization rules for all these different dynamical processes on networks.

So far the dynamics on extensive systems composed of many coupled identical units has been extensively studied, for example, the complete synchronization in coupled chaotic systems based on the stability analysis of the synchronous manifold and in the framework of the master stability function [17]–[23]. However, the assumption of identical units is not very realistic for many realistic systems, in which the units composing the ensemble always present a disparity in the values of some characteristic parameters. Under these conditions, the system (parameter) diversity becomes very important [24]–[29]. It is, however, still unknown how these nodes with different characteristic parameters settled on networks determine the systems dynamics, especially when some key parameters are properly placed on some special positions of networks. This optimal configuration problem has never been carefully studied, to the best knowledge of the authors, although it certainly could help to better understand the collective behaviors of coupled nonidentical systems, which are determined by not only the interplay between the temporal information (the node dynamics with different parameters) and the spatial information (these nodes' positions on networks), but also their matchings.

For this purpose, we study the optimal configuration problem for the vibration spectrum (or frequencies) of coupled nonidentical harmonic oscillators on complex networks. Each vertex is occupied by a mass point and they are connected by an edge (spring) each other with a uniform spring constant. This mass-spring (linear) model and the normal mode analysis originally developed in classical mechanics [30], [31], have been found very useful in many other disciplines, such as lattice vibrations and associated phonon excitations in solid-state physics [32], and protein dynamics in structural biology [33], [34]. As we are interested in the diversity effect, we only consider the case for each node having a different mass; this is the key distinction with all previous studies on coupled harmonic oscillators [35]–[38].

## Model and Results

### Model

The motion equation for the systems can be written as
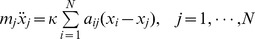
(1)where 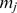 represents the *j*-th oscillator's mass, *κ* is the coupling strength (or spring coefficient), and *N* represents the number of mass points. 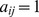 if nodes *i* and *j* are connected and 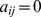 otherwise. For simplicity, we take 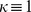 throughout the paper. The masses 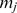 for all *j* are different. The system's motion can be viewed as *N* normal modes with different frequencies: 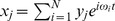, where 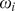 and 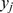 denote one independent frequency and the amplitude, respectively. 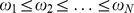. Clearly 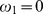, representing the translational motion. For the other modes, usually the second smallest (slowest) vibration frequency 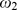 characterizes the most global vibrational motion of the systems, whereas the largest vibration frequency 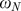 reflects the most tightly packed and constrained local motion; both of them are the most representative. Therefore, below we only consider the values of 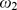 and 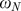 for different spatial configurations of the masses.

The above equations [Eqs. (1)] can be written in a compact form based on the normal mode analysis:
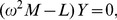
(2)where the mass matrix 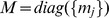, the Laplacian matrix 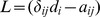, where 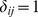 for *i* = *j* and 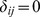 otherwise, 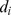 is the degree of node *i*, and *Y* is the *N*-dimensional nonzero column vector with the components 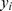.

Solving Eqs. (2) is equivalent to finding *ω* satisfying 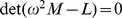, where “det” denotes the determinant. Since *M* is nonsingular, it is convenient to change the mass-weighted Laplacian matrix to a symmetric one and get a faster numerical result. Therefore, we have
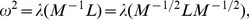
(3)where *λ* denotes the eigenvalue of the corresponding matrix. And we obtain *ω* based on solving the eigenvalues of the symmetrical matrix 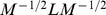 for a given network. In numerics, we have studied various networks, such as the random Erdos-Renyi (ER) [39], scale-free (SF) [40], and small-world (SW) [41] networks. For each network, an ensemble of masses is produced from a random distribution (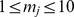) and these values are fixed and scattered on networks. For each configuration, its vibration frequencies can be easily calculated from the above eigenvalue analysis [Eq. (3)]. We are interested in how the correlation between degrees and masses influences the normal mode frequencies 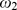 and 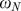.

### Simulation results

**Figure 1:**
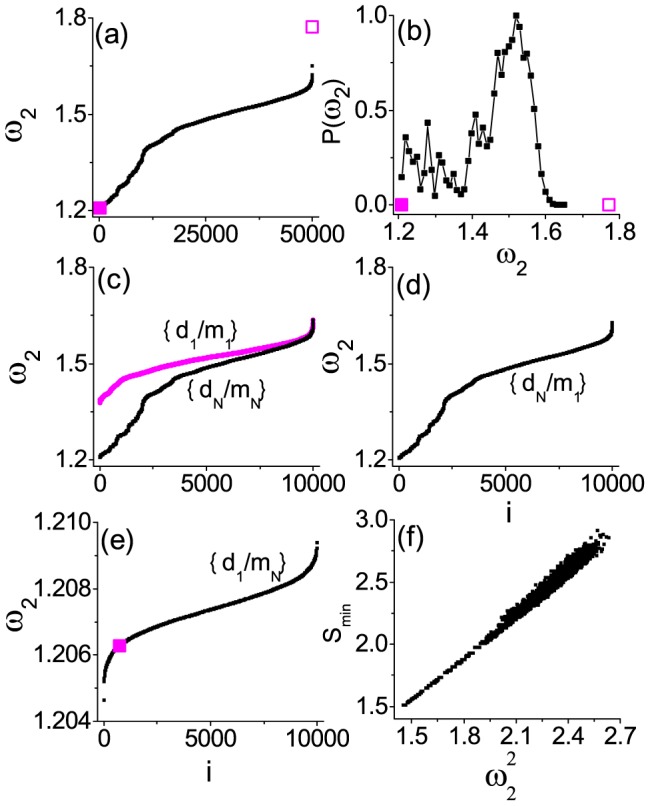
The results of 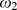 for a random ER network. *N* = 200, 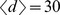, 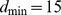, and 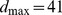, the plot includes completely random configurations (black points), the point-to-point-positive correlation configuration (open squares), and the point-to-point-negative correlation configuration (solid squares) in (a); the single-point-positive correlation configurations 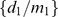 and 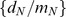 in (c); and the single-point-negative correlation configurations 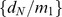 in (d) and 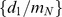 in (e). (b) The histogram for random configurations. (f) 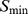 vs 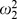. A remarkable finding in (e) is 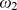
's are not only very small, but also their range is very narrow, indicating one is enough in determining the networked dynamics. For more details, see the text.

Figure 1(a) illustrates the numerical results of 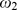 in an ascending order for completely random configurations (i.e., all masses can be arbitrarily shuffled on complex networks); a random ER network (*N* = 200 and the average degree 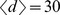) is chosen here. The corresponding distribution re-scaled by the peak value is given in Fig. 1(b). Clearly 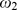 shows a very wide distribution from 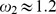 to 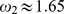. However, we may also choose configurations purposefully, such as point-to-point-positive correlation between the node masses and the node degrees (namely, we may arrange the masses in an ascend order 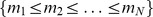 and the degrees in an ascend order also 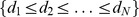, and put these arranged masses on the network in a point-to-point-positive correlation order 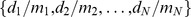). We really find its 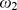 for this configuration is maximal, as shown the open square in Figs. 1(a) and 1(b). Correspondingly, for the configuration of point-to-point-negative correlation 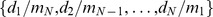 illustrated by a solid square, it seems that 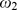 is very small.

Below let us see if the above strong correlation condition for the point-to-point matching could be loosened. Figure 1(c) shows the results for the single-point-positive correlation 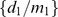 (upper curve) and 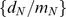 (lower curve), with all other 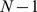 masses randomly placed. Here we use 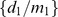 to denote the configuration of putting the lightest mass on the lowest-degree node, whereas 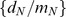 for that of the heaviest on the highest-degree node. It is discernible that the curve for 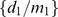 is slightly above the counterpart for 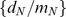, which is quite similar to that for the random configurations in 1(a). Moreover, Figs. 1(d) and 1(e) show the results for the single-point-negative correlation 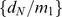 and 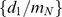, respectively, with all other 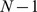 masses randomly placed again. Clearly the distribution in 1(d) is still similar to that for the random configurations in 1(a). However, very remarkably the values of 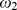 for 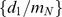 (with one and only one heaviest mass on the lowest degree) have been greatly squeezed into a much smaller regime: 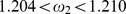 and they are indeed very small [noticing the different ordinates and their scales used in Figs. 1(a) and 1(e)]. This range is only one-hundredth of the original range for the random distribution or other single-point matching distributions. We also find that 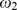 for the point-to-point-negative correlation in Fig. 1(a) is not minimal, which is re-plotted in Fig. 1(e) with the same solid square. This indicates that the single-point matching 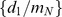 has already caught the essential feature of the collective behavior in such a model of harmonic oscillators on random networks, regardless of all other nodes. As a result, for 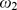, the single-point matching 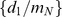 should give rise to 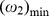, whereas the point-to-point matching 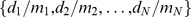 should lead to 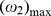, although these observations still need proof in theoretical analysis.

**Figure 2:**
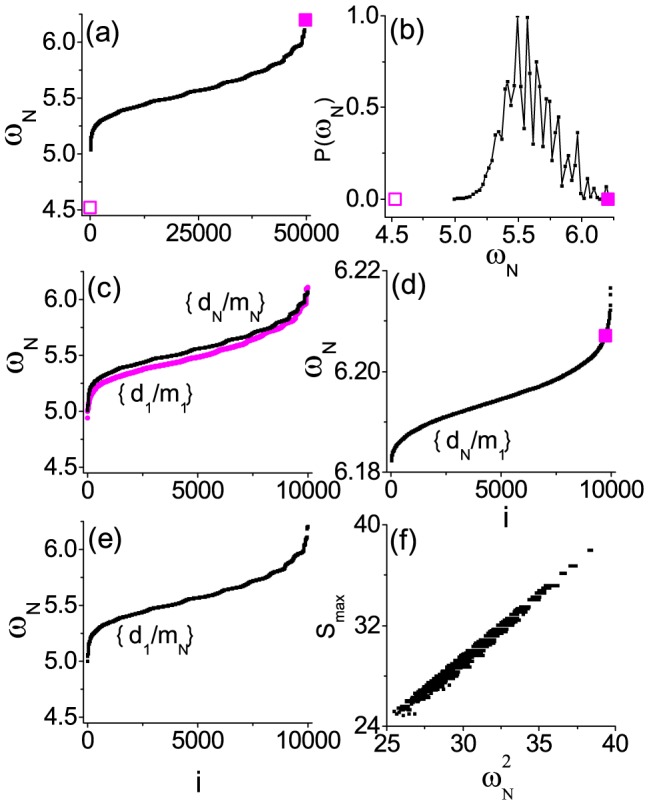
The results of 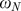 for a random ER network. Similar to Fig. 1 for the results of 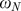 with the same ER network considered instead. Again the effect of one is enough appears, but this time 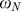 becomes maximal for the single-point-negative correlation configuration 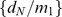 in (d). For more details, see the text.

Figure 2 presents the results for 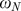 instead with the same random network and the ensemble of masses. For the random configurations, 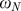
's are distributed in a very wide range (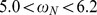). The configurations for the point-to-point-positive correlation and the point-to-point-negative correlation, as shown the open and solid squares in Fig. 2(a), give rise to the minimal and maximal values of 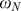, respectively. They are just opposite to those for 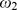. Again 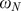 for the configuration of point-to-point-positive correlation is very far from the distribution in 2(b). In Figs. 2(c) and 2(e), the single-point matching distributions for 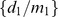, 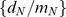, and 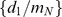 give no significant difference with that for the random distribution. However, for the single-point-negative correlation 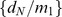 in 2(d), we do find 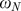
's have been squeezed into a much smaller regime; this time it corresponds to the part of the largest 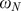. All these findings show the key qualitatively unchanged features with those for 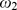.

**Figure 3:**
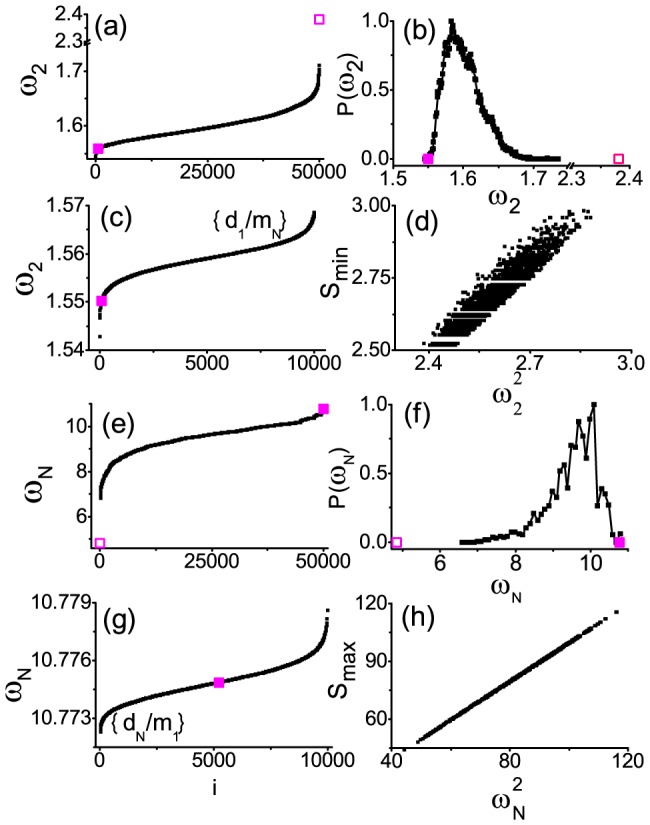
The results of 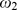 and 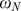 for a scale-free network. Similar to Figs. 1 and 2 for a scale-free network (*N* = 200, 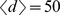, 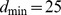, and 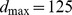) instead.

To show the generality of matching rules for any random networks, we give some more examples. For instance, the results for a scale-free network (*N* = 200 and 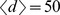) with a randomly distributed ensemble of masses are shown in Figs. 3(a)–3(h). Figure 3(a) illustrates 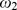 for random configurations with the two extreme points (open and solid squares) for the configurations (point-to-point-positive and point-to-point-negative correlations). The histogram is shown in 3(b). Again Fig. 3(c) exhibits the unusual effect of single-point-negative correlation 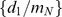 for 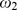, indicative of an insensitivity of vibration frequency if the temporal information and spatial information of only one node are matched. Similarly, Figs. 3(e)–3(h) give the patterns for 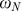, showing the same qualitative results as in Fig. 2.

**Figure 4:**
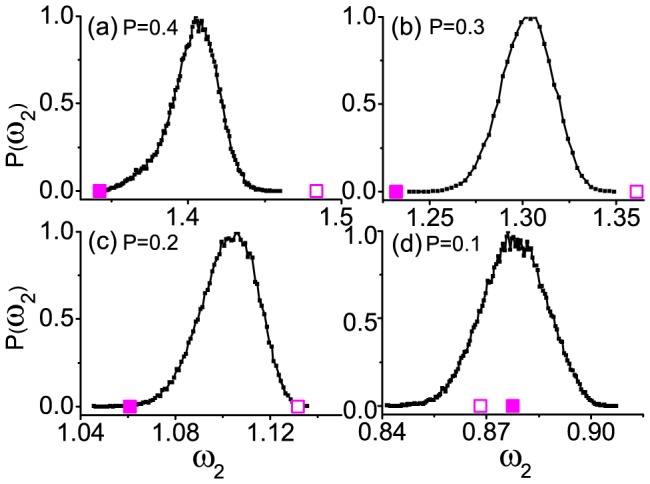
Histograms of 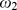 for random configurations of a SW network. (a)–(d) *N* = 200 and 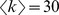 with different rewiring probabilities *P*: *P* = 0.4, 0.3, 0.2, and 0.1, respectively. The open and solid squares represent the configurations of point-to-point-positive and point-to-point-negative correlations between the node masses and node degrees, respectively.

Figures 4(a)–4(d) illustrate the distributions of 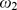 for random configurations of a SW network with some different rewiring probabilities *P*: *P* = 0.4, 0.3, 0.2, and 0.1, respectively; *N* = 200 and 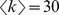. The open and solid squares represent the configurations of point-to-point-positive and point-to-point-negative correlations between the node masses and node degrees, respectively. Clearly with increasing random connections for larger *P*, such as the plots in Figs. 4(a) and 4(b), the two points keep as extreme, locating far away the distribution for random configurations. However, with decreasing random connections for smaller *P*, as shown in 4(c) and 4(d), these relations become broken. Based on these comparisons, we understand that the rules of point-to-point matching and the single-point matching may only work for sufficiently random networks, such as ER or SF networks, and these rules could gradually be broken with the weakening of the network random connections, such as the SW networks.

## Analysis

So far some unusual effects of matching rules have been well revealed. Below let us demonstrate them in a rigid way with the aid of mathematical analysis. As we have known, the normal mode frequencies are solely determined by the mass-weighted Laplacian matrix [Eq. (3)]. Therefore, some approximation results for the weighted Laplacian matrix developed in the matrix algebra and applied in the chaos synchronization study should be very valuable for our problems here [20], [21], [42]; for the second smallest and largest eigenvalues 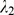 and 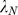, we have
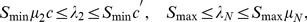
(4)where *c* and 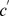 can be approximated by 1 for most complex networks, and 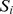, the intensity function of a node, is defined as 
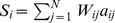
, where 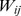 is the element of the weighted matrix. Since 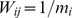 here, we have 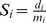 and further
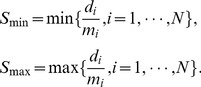
(5)


When the network is sufficiently random (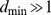 and 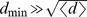),
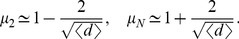
(6)Therefore, for a sufficiently large 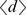,
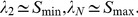
(7)As a result, 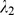 and 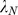 (and hence 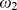 and 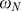 correspondingly) should be completely determined by 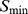 and 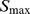, respectively.

These linear relations have been perfectly proved by our simulations, for example, the plot of 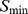 versus 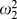 in Fig. 1(f) and that of 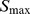 versus 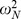 in 2(f). Based on these analyses [Eq. (5)], we immediately know that the configuration of single-point-negative correlation 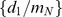 (with the heaviest mass on the lowest-degree node) should always give rise to 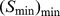 and 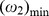 irrespective of all other masses. Here one node match is dominant.

The result for point-to-point-positive correlation for 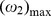 can also be easily understood. Without losing generality, suppose that the masses and the degrees have been perfectly placed, i.e.,
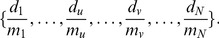
(8)Here 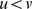, 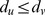 and 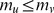. If any one pair of nodes (*u*-th and *v*-th) are not placed in order, namely, node with mass 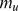 is placed on the node of degree 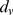 while 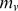 on 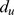 instead, we have the new configuration
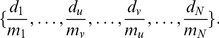
(9)


Based on the following inequalities
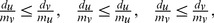
(10)giving rise to
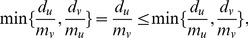
(11)we can derive that any one permutation from the original, perfect configuration of the point-to-point-positive correlation [Eq. (8)] would not let 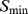 increase. Therefore, the original configuration would be globally optimal, corresponding to 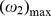. On the other hand, we may use the same idea to the analysis of 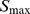 for 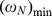.

**Figure 5:**
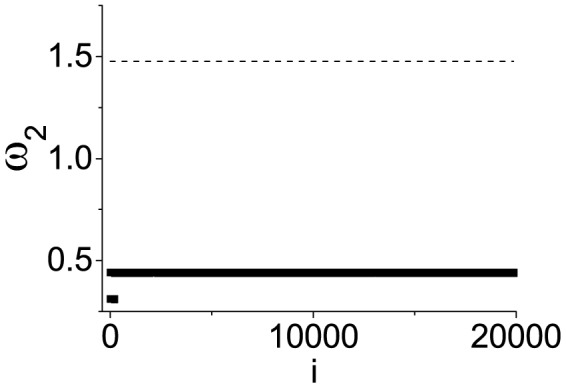
Controllability of network. Control of 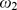 by adding one node (*m* = 10) and connecting it to any one node (lower part) and any two nodes (higher part) in an ER network, the same as in Figs. 1 and 2, in contrast to the uncontrolled value of 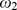 (dashed line).

Finally after mastering these features of coupled harmonic oscillators, we may even control the systems collective behavior by a slight manipulation [43]–[45]. E.g., a single node with the heaviest mass *m* = 10 (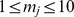) has been added and connected to any nodes of an ER network, as shown in Fig. 5, where the lower part represents *N* trials with one connection and the higher part represents *Z* (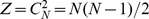) trials with any two connections; *N* = 200. Therefore, we have 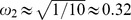 for the lower value and 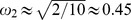 for the higher value. In contrast, the original big value 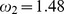 is illustrated by a dashed line. By this method, we may accomplish precise control of 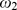. Similar results have also been obtained for other networks.

## Discussion

In conclusion, we have studied the parameter diversity effect of coupled harmonic oscillators with different masses on complex networks, and found that the values of 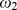 and 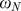 highly depend on the configurations of these masses on the network spatial structure. Especially, two key matching rules including the point-to-point matching and single-point matching determine their extreme values. These findings might be helpful for explaining some interesting phenomena, such as biological swarming and flocking in nature, where the match of the head's ability with its position in hierarchy is always crucial for the behaviors of the whole group. In addition, the optimal configuration not only exhibits the importance of matching between the node dynamics and the node position and their impact on the collective behaviors of coupled systems, but also provides a potential control method for the manipulation of the systems dynamics. A possible candidate for this might be the point mutation in the protein dynamics study. We expect that the feature of “one is enough” may arouse general interest in studying the structure-dynamics-function relation in complex systems, and the matching rules may also become the common organization rules for various dynamical processes on complex networks.
